# Altered TIMP-3 Levels in the Cerebrospinal Fluid and Plasma of Patients with Alzheimer’s Disease

**DOI:** 10.3390/jpm12050827

**Published:** 2022-05-19

**Authors:** Jung Hyun Park, Sun-Jung Cho, Chulman Jo, Moon Ho Park, Changsu Han, Eun-Joo Kim, Gi Yeong Huh, Young Ho Koh

**Affiliations:** 1 Department of Chronic Disease Convergence Research, Division of Brain Disease Research, Korea National Institute of Health, 187 Osongsaengmyeong2-ro, Osong-eup, Heungdeok-gu, Cheongju-si 28159, Korea; ntpeace@naver.com (J.H.P.); sjcho225@korea.kr (S.-J.C.); ironman@korea.kr (C.J.); 2 Departments of Neurology, Korea University Medical College, Ansan Hospital, 123 Jeokgeum-ro, Danwon-gu, Ansan-si 15355, Korea; parkmuno@korea.ac.kr; 3 Departments of Psychiatry, Korea University Medical College, Ansan Hospital, 123 Jeokgeum-ro, Danwon-gu, Ansan-si 15355, Korea; hancs@korea.ac.kr; 4 Department of Neurology, Pusan National University Hospital, 179 Gudeok-ro, Seo-gu, Busan 49241, Korea; eunjookim@pusan.ac.kr; 5 Department of Forensic Medicine, Pusan National University School of Medicine, 49 Busandaehak-ro, Mulgeum-eup, Yangsan-si 50612, Korea; gyhuh@pusan.ac.kr

**Keywords:** TIMP-3, CSF, Plasma, Alzheimer’s disease

## Abstract

Tissue inhibitor of metalloproteinase-3 (TIMP-3) is a component of the extracellular environment and is suggested to play an indirect role in regulating Aβ production and the pathophysiology of Aβ deposition in brains. However, studies on the amount of TIMP-3 in bodily fluids of Alzheimer’s disease (AD) patients have not been conducted. Here, we investigated the relationship between fluid TIMP-3 levels and AD pathology. We first showed that the fluid levels of TIMP-3 were lower in AD dementia patients compared with in non-AD patients. ELISA results revealed that plasma levels of TIMP-3 in 65 patients with AD were significantly lower than those in 115 healthy control subjects and 71 mild cognitive impairment (MCI) subjects. Furthermore, we found that cerebrospinal fluid (CSF) level of TIMP-3 was decreased in AD compared with that in healthy control. These data suggest that fluid TIMP-3 levels negatively correlated with progress of cognitive decline. Collectively, our study suggests that alterations of fluid TIMP-3 levels might be associated with AD pathology.

## 1. Introduction

Alzheimer’s disease (AD) is a progressive neurodegenerative disease in which cognitive impairments are typically preceded by extracellular deposition of characteristic diffuse and insoluble plaques in the brain [1,2]. It is also one of the most common types of dementia and an important cause of mortality in elderly persons [3]. Since the major pathological hallmark of AD is the formation of neuritic plaques composed of amyloid-β (Aβ) peptides, clinical studies targeting Aβ have been reported over the past decades.

In recent years, metalloproteinases (MMPs) and a disintegrin and metalloproteinases (ADAMs) have been found to play a role in AD [4,5]. For instance, ADAM-10 and ADAM-17 have been reported to lead to increasing secretion of soluble amyloid precursor protein (APP) fragments and reduction of Aβ generation [6]. A recent study reported that fluid levels of ADAM-10 are increased in AD patients [7]. MMP-9 also has been extensively studied because of its potential role in amyloid clearance. It has been already reported that MMPs are elevated in the postmortem brains of AD patients [8,9].

Tissue inhibitors of metalloproteinases (TIMPs) are proteins that play an important role because they reversibly inhibit enzymes such as the zinc protease superfamily, predominantly MMPs and ADAMs [10]. Since altered regulation of MMPs can result in cancer, inflammatory diseases, and degenerative diseases [11,12,13,14], studies targeting TIMPs have also reported that TIMPs widely influence various disease pathologies. Many studies demonstrate that TIMPs can be used as biomarkers for various diseases including cancer and strokes [15,16,17]. Furthermore, plasma and CSF levels of MMPs (MMP-1, MMP-2, and MMP-9) and TIMPs (TIMP-1 and TIMP-2) were altered in various types of dementia [8,18,19].

Among the TIMP family, TIMP-3 is the only extracellular matrix (ECM)-bound TIMP [20], and it has broadest range of substrates, including all MMPs. Furthermore, TIMP-3 is the only member of the TIMP family that is capable of inhibiting ADAM-10 and ADAM-17 [21,22]. Therefore, TIMP-3 plays an indirect role in regulating the amyloidogenic pathway and Aβ production. Recent research indicates that TIMP-3 protein levels were increased in human AD brain and APP transgenic mice, suggesting that increased levels of TIMP-3 in AD may contribute to Aβ accumulation [23]. There have also been reports of a significantly increased TIMP-3 in aged/CAA leptomeningeal arteries, which indicate that regulation of the ECM is involved in the pathophysiology of Aβ deposition in the cerebral vessels [24]. Although many clinical studies have been conducted on the associations with various diseases by targeting fluid levels of TIMPs, the relationship between TIMP-3 and AD still remains unknown. In a previous study, we reported that the plasma levels of vascular growth factor receptor 2 (VEGFR-2) were altered in patients with AD [25]. As a major VEGFR-2 signal regulator, TIMP-3 has been reported to inhibit angiogenesis by decreasing VEGF-VEGFR-2 interaction [26].

Therefore, we hypothesized that TIMP-3 fluid levels may be altered in patients with AD. As little is known about the clinical significance of plasma levels of TIMP-3, we screened TIMP-3 levels in the plasma of subjects with mild cognitive impairment (MCI) as well as in AD patients and compared them with healthy controls. Furthermore, we evaluated whether TIMP-3 levels were altered in the CSF of patients with AD. Fluid TIMP-3 levels were negatively correlated with the progression of AD. These results highlight the importance of TIMP-3 as a potent biomarker for AD.

## 2. Material & Methods

### 2.1. Human Blood Samples

The subjects, including healthy controls, MCI, and dementia, participated in this study, designated from the population-based Ansan Geriatric (AGE) cohort, which was established to study common geriatric diseases in elderly Koreans aged 60 to 84 years [27,28,29]. The sampling protocol and design of the AGE study have been previously described [28,30]. Each patient with dementia met the criteria for the Diagnostic and Statistical Manual of Mental Disorders, fourth edition. Subjects with cognitive and memory impairment were assessed using the Korean version of the Consortium to Establish a Registry for Alzheimer’s Disease (CERAD-K) neuropsychological battery as previously described [31]. All dementia patients met the criteria for probable AD, as established by the National Institute of Neurological and Communicative Disorders and Stroke and the Alzheimer’s Disease and Related Disorders Association (NINCDS-ADRDA). The diagnosis of MCI was based on the Mayo Clinic criteria as previously described [32,33]. In total, blood samples from 251 subjects were collected, and the demographic and clinical variables of each participant are shown in Table 1. This study was approved by the Institutional Review Board (IRB) of the Korea Disease Control and Prevention Agency (KDCA) with approval number (2016-02-22-P-A, 2017-05-05-P-A, 2020-03-04-P-A). All the processes in this study were performed following the relevant guidelines and regulations.

### 2.2. CSF Sampling

Human CSF was obtained from and provided by the Pusan National University Hospital Brain Bank (PNUHBB). Each subject signed an informed consent form before their inclusion. All procedures were performed according to the relevant guidelines and regulations [34]. All participants were examined by a neurologist who specialized in neurodegenerative diseases, followed by a clinical interview, and a neurological examination [35]. Patients with MCI met both the NIA-AA core clinical criteria for MCI and the modified Petersen’s criteria [36]. All patients with AD dementia (ADD) satisfied the NIA-AA core clinical criteria for probable ADD [37].

For CSF collection, CSF samples from 30 subjects were collected as previously described [34]. CSF samples were analyzed at the Research Institute for Convergence of Biomedical Science and Technology at Pusan National University Yangsan Hospital (PNUYH). CSF Aβ_1-42_, total Tau, and pTau_181_ levels were measured with the INNOTEST ELISA kit (Fujirebio Diagnostics, Ghent, Belgium) following the manufacturer’s instructions [34].

### 2.3. Cell Cultures

Human iPSC-derived neuronal cells were obtained from Axol Biosciences (Little Chesterford, UK). The cells were differentiated into cerebral cortical neurons in approximately 7 days following the manufacturer’s protocol. Cells were cultured in a humidified atmosphere of 5% CO_2_ at 37 °C.

### 2.4. Animals

APPsw/PS1ΔE9 transgenic mice were used for this study, as previously reported [38]. All experiments were approved by the Institutional Animal Care and Use Committee (IACUC) of the KDCA and conducted in accordance with the guidelines for the care and use of laboratory animals by the KDCA.

### 2.5. Antibodies and Reagents

The anti-TIMP-3 antibody (AB6000) was purchased from EMD Millipore (Darmstadt, Germany). Anti-β-actin (A5316) antibody was purchased from Sigma-Aldrich (St. Louis, MO, USA). Anti-Aβ (6E10, SIG39300) was purchased from Convance (Denver, PA, USA). Alexa Fluor 488 goat anti-mouse IgG and Alexa Fluor 594 goat anti-rabbit IgG were obtained from Molecular Probes (Eugene, OR, USA).

### 2.6. Measurement of Blood Proteins by ELISA

All plasma samples were aliquoted and stored at −80 °C until assayed collectively by an investigator who was blinded to patient assignment. The levels of TIMP-3 were determined by the ELISA kit (USCN, Wuhan, China) following the recommended manufacturer’s protocol.

### 2.7. Western Blotting

The protein concentrations were measured with Bradford method (Bio-Rad, Hercules, CA, USA) protein assays following the manufacturer’s protocol. Proteins were resolved by 4–12% NuPAGE gel (Invitrogen) and transferred onto nitrocellulose membranes. Membranes were blocked in TBS with 5% nonfat dry milk and 0.1% Tween 20 and incubated with the primary antibody at 4 °C for overnight and washed. Membranes were incubated with horseradish peroxidase (HRP) anti-rabbit and anti-mouse secondary antibody. Protein bands were detected with a chemiluminescence kit (Amersham Pharmacia Biotech, Buckinghamshire, UK)

### 2.8. Real-Time Reverse Transcription Polymerase Chain Reaction

Real-time quantitative polymerase chain reaction (RT-PCR) analysis was performed with SYBR Green PCR core reagent in a two-step RT-PCR protocol according to the manufacturer’s protocol (Applied Biosystems, Warrington, UK). The primer sequences for the RT-PCR experiments were as follows: TIMP-3 sense 5′-CAAGATGCCCATGTGCAGT-3′ and antisense 5′-GCCATCATAGACGCGACCTG-3′. GAPDH sense 5′-CAGCCTCAAGATCATCAGCA-3′ and antisense 5′-TGTGGTCATGAGTCCTTCCA-3′. The relative TIMP-3 level was normalized to the GAPDH levels. PCR reactions were performed using ABI Prism 7900 SDS (Applied Biosystems, Warrington, UK).

### 2.9. Immunofluorescence Assay

Anesthetized mice were perfused with PBS, and tissues were harvested rapidly. Obtained brain tissues were fixed with 4% paraformaldehyde and then transferred to 30% sucrose solution. OCT compound-embedded brains were sectioned to 20 μm and mounted on glass slides. For the immunohistochemical analyses, each brain section was permeabilized with 0.03% Triton X-100 in PBS, blocked with 5% normal goat serum at room temperature, and incubated with the appropriate primary antibodies overnight. The next day, sections were washed and incubated with secondary antibodies. Labeled brain sections were visualized by fluorescence microscope (Zeiss, Germany).

### 2.10. Statistical Analyses

The results are expressed as mean ± SD and mean ± SEM. The Mann-Whitney U-test and the Kruskal-Wallis test were used to analyze the demographic and clinical variables within groups. Correlation between factors was checked using Spearman’s method. Statistical analyses of the present study were performed using SPSS 12.0 (IBM, Armonk, NY, USA). A *p* value less than 0.05 was considered statistically significant.

## 3. Results

**Figure 1:**
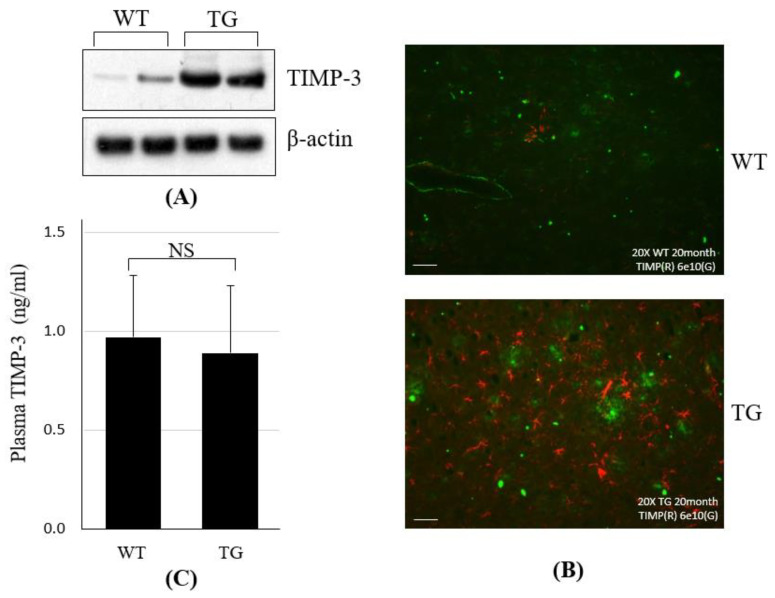
(**A**) Immunoblot analysis for TIMP-3 in the cerebral cortex of wild-type (WT) and APP Swedish/PS1dE9 transgenic (APP Tg) mice. β-actin was used as a loading control. (**B**) Detection of TIMP-3 in the parietal cortex of AD mice brains. Immunostainings of anti-TIMP-3 (Red) and anti-Aβ_1-42_ (Green) in the brain of WT and APP Tg mice (scale bar = 50 μm). (**C**) The plasma TIMP-3 levels of WT (*n* = 4) and TG (*n* = 4) were measured by ELISA assay. Each value is presented as the mean ± SD. TG, Transgenic; NS, not significant; TIMP-3, Tissue inhibitor of metalloproteinase-3; SD, Standard deviation.

We previously reported that TIMP-3 expression is increased by Aβ in vitro [25]. In particular, because TIMP-3 levels are elevated upon Aβ treatment, we wondered whether TIMP-3 expression was also altered in an AD mice brain. As shown in Figure 1A, we confirmed the increase of TIMP-3 in brain cortex regions of 20-month-old APP Swedish/PS1delE9 Tg (APP Tg) mice. To assess changes in TIMP-3 expression in AD mice, we also performed immunostaining with anti-TIMP-3 and anti-Aβ (6E10) in WT and APP Tg mice. We found that the deposition of TIMP-3 was enhanced in APP Tg mice (Figure 1B). Furthermore, we also monitored the plasma TIMP-3 levels in WT and APP Tg mice. The plasma concentration of TIMP-3 was decreased but not significantly in APP Tg mice compared with in WT mice (Figure 1C).

**Figure 2:**
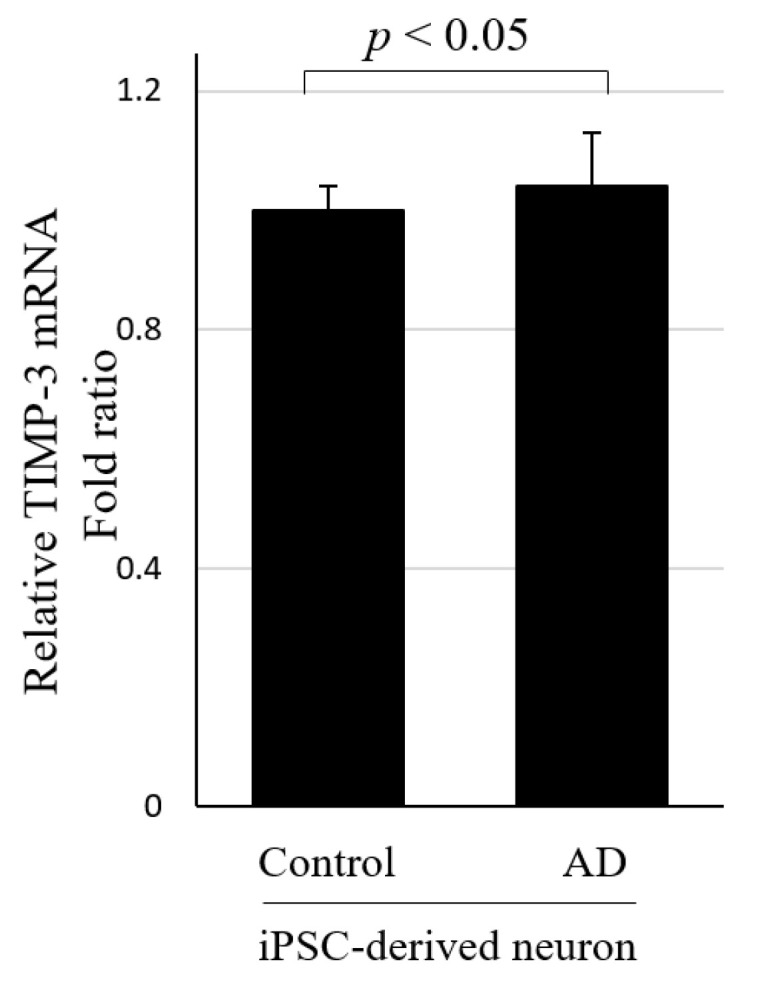
Relative TIMP-3 mRNA levels in human iPSC-derived neuronal cells from an AD patient (*n* = 3) and a control (*n* = 3). Results are expressed as mean ± SD; statistical significance is indicated by *p* < 0.05. AD, Alzheimer’s Disease; SD, Standard deviation.

We then examined the TIMP-3 expression in human iPSC-derived neuronal cells from AD patients. Once the cells were differentiated to neurons, the TIMP-3 mRNA levels were slightly increased in AD patients compared with healthy controls (Figure 2).

Next, we investigated whether elevated TIMP-3 is correlated with bodily fluid levels. We first compared the levels of TIMP-3 in the plasma from 251 subjects with dementia, subjects with MCI, and healthy controls. The demographic and clinical variables of participants are shown in Table 1. The mean age of the normal control participants was 71.9 ± 0.43 years, and it was 73.05 ± 0.54 years for the subjects with MCI. The mean age of the dementia participants was 75.1 ± 0.75 years.

**Figure 3:**
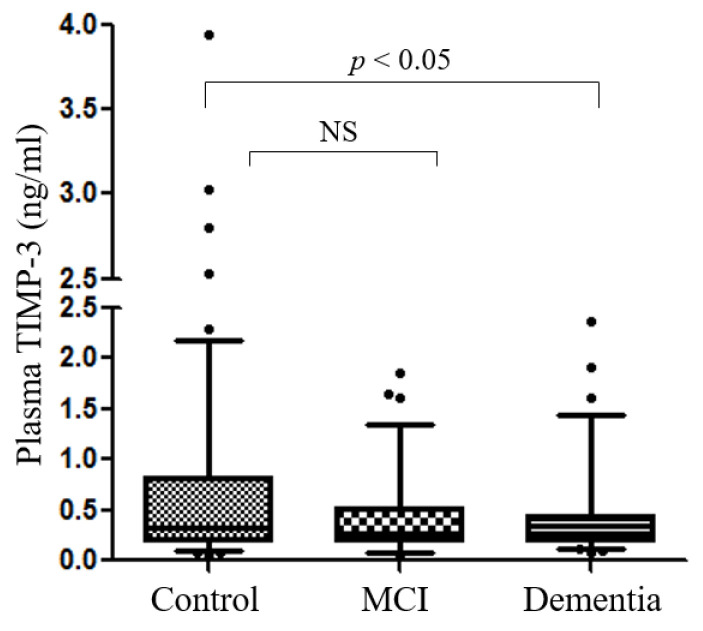
Analysis of TIMP-3 levels in human plasma. Plasma TIMP-3 concentrations were measured by ELISA. The differences in the relative amounts of TIMP-3 were compared between dementia, MCI, and controls using the Mann-Whitney U-test within different groups. NS = not significant.

Dementia participants were less educated than the normal control participants. The MMSE score was lower in dementia groups but in the normal range in the control and MCI groups. Interestingly, the plasma concentrations of TIMP-3 were significantly lower in the dementia group (0.39 ± 0.05 ng/mL) compared with the control group (0.6 ± 0.06 ng/mL) in the Mann-Whitney U-test results (*p* = 0.029) (Figure 3). Analysis of clinical characteristics showed in Table 2 that plasma TIMP-3 levels were negatively correlated with CDR scores (r = −0.153; *p* = 0.015). Plasma TIMP-3 levels were also negatively correlated with LDL cholesterol (r = −0.142; *p* = 0.025) and glucose (r = −0.127; *p* = 0.046).

We also investigated the CSF levels of TIMP-3 to clarify whether fluid levels of TIMP-3 are related to clinically overt ADD. Patient characteristics are presented in Table 3.

**Figure 4:**
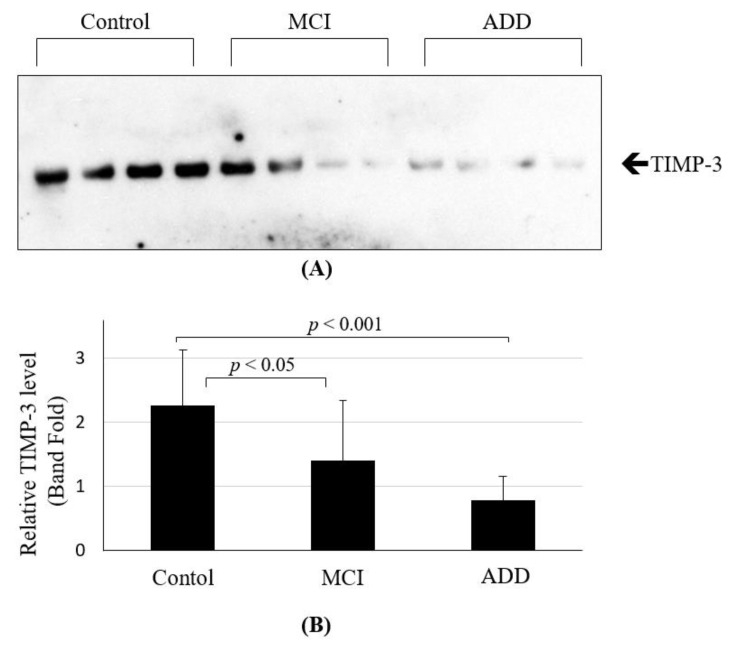
(**A**) Levels of TIMP-3 in human CSF were estimated by immunoblot analysis. The CSF from healthy control (*n* = 10), MCI (*n* = 10), and ADD (*n* = 10) w probed with an anti-TIMP-3 antibody. (**B**) Relative expression levels of TIMP-3 are represented as mean ± SD, and statistical significance is indicated by *p* < 0.05 and *p* < 0.001. Mild cognitive impairment (MCI); Alzheimer’s Disease Dementia (ADD).

Patients with ADD were older than the control and MCI groups. The mean age of the normal control participants (3 males, 7 females) was 63.8 ± 3.8 years, an in patients with MCI (3 males, 7 females), it was 63.6 ± 3.7 years; in patients with ADD (5 males, 5 females), it was 68.1 ± 3.3 years. Furthermore, concentrations of Aβ_1-42_, total Tau, and phosphorylated-Tau (pTau) were measured with ELISA. As expected, the CSF levels of Aβ_1-42_ were different among the three groups (*p* < 0.001; Kruskal-Wallis test). The CSF Aβ_1-42_ concentrations were lower in patients with ADD (401.3 ± 27.4 pg/mL) and MCI (766.3 ± 70.9 pg/mL) compared with the control subjects (1030.1 ± 32.6 pg/mL). The CSF pTau concentrations were higher in ADD subjects (81.5 ± 9.3 pg/mL) compared with control subjects (50.3 ± 2.4 pg/mL) and MCI subjects (41.2 ± 4.5 pg/mL) (Table 3). Immunoblot analysis revealed the CSF levels of TIMP-3 in the control, MCI, and ADD groups (Figure 4A). Levels of TIMP-3 were lower in the CSF of ADD patients compared with MCI patients and healthy controls (Figure 4B). We then investigated the relationships between TIMP-3 levels and other biomarkers, such as Aβ_1-42_, total Tau, and pTau in the CSF (Table 4). We observed positive correlations between TIMP-3 and Aβ_1-42_ levels in CSF (*r* = 0.515, *p* = 0.004). Moreover, TIMP-3 levels were also negatively correlated with pTau levels (*r* = −0.372, *p* = 0.047). Overall, these results suggest that CSF TIMP-3 is likely to be associated with Aβ and pTau, which indicate pathological progression of the disease.

## 4. Discussion

TIMP-3 is a component of the extracellular environment that plays diverse roles, including in matrix regulation, inflammation, angiogenesis, and potentially the pathogenesis of AD through indirect mechanisms [22]. The expression of TIMP-3 in AD has not been fully elucidated, and to the best of our knowledge, this is the first study investigating plasma and CSF levels of TIMP-3 in diagnostic groups (control, MCI, ADD). Notably, our results reveal that TIMP-3 levels in AD decreased not only in plasma but also in CSF. These results suggest that a change in the fluid levels of TIMP-3 may indicate AD progression. Previous studies have shown that TIMP-3 expression was increased in the brain of an AD mouse model and in a human AD brain [23]; the higher levels of TIMP-3 may thus contribute to increased conversion of APP to Aβ, contributing to the pathogenesis of AD. Therefore, we expected fluid TIMP-3 levels might be increased. However, our result showed that TIMP-3 fluid level is lower in AD patients.

TIMP fluid levels have been reported in patients with various diseases, but the levels of TIMPs are different depending on the disease condition. High levels of blood MMP-9 and TIMP-1 indicate worse prognoses in lung cancer [39] and breast cancer [40]. High levels of TIMP-1 have been found in infracted brain tissue [41] and in the blood of ischemic stroke patients [42]. Plasma TIMP-3 levels were lower in patients with oral squamous cell carcinoma [43] and higher among subjects who developed acute respiratory distress syndrome [44]. The serum levels of MMP-3 and TIMP-3 correlate with survival in ovarian cancer [45]. In AD patients, it has been shown to low CSF TIMP-1 levels [46]. Interestingly, ADAM-17 activity is increased in the CSF and plasma of AD patients [29]. In the non-amyloidogenic process, APP is cleaved by ADAM-17 as an α-secretase to release soluble N-terminal fragments of APP (sAPPα) and C83 fragments [47]. A recent study shows that ADAM-17 levels are elevated in the brains of patients with AD and have been shown to localize in amyloid plaques [48]. Considering the role of TIMP-3 as an inhibitor of ADAM-17, our result is interesting that TIMP-3 levels are increased in both in vitro and in vivo AD model but lower in the CSF and plasma of AD patients. Our findings also imply that increased ADAM-17 activity might be linked to the decrease of TIMP-3 in the CSF or plasma of AD.

Recent research indicated that TIMP-3 is also increased in the walls of the blood vessels of CAA [24], which commonly coexists in patients with AD [49,50,51]. Proteomic profiling of isolated leptomeningeal vessels of CAA identified enriched proteins, including TIMP-3 and clusterin [24]. Considering that amyloid deposits are frequently observed in brain vessels in AD, TIMP-3 might be involved in amyloid deposits in brain vessels. Moreover, elevated TIMP-3 was also observed in brain vessels of cerebral autosomal dominant arteriopathy with subcortical infarcts and leukoencephalopathy (CADASIL) [52,53]. TIMP-3 forms complexes with NOTCH-3 and accumulates in the extracellular matrix of brain vessels of patients and mice with CADASIL, an inherited cerebral small vessel disease and a cause of stroke and dementia [52]. Given that TIMP-3 was co-localized with NOTCH-3 deposits in CADASIL [52], the recruitment of TIMP-3 to Aβ or NOTCH-3 aggregates may affect ADAM-17-mediated APP cleavage and contribute to AD pathology. Our observations imply that the reduced fluid levels of TIMP-3 in patients with AD might be associated with aggregates of TIMP-3 and Aβ.

We previously showed that Aβ reduced VEGFR-2 levels and increased TIMP-3 in endothelial cells [25]. It was reported that TIMP-3 reduced VEGF-mediated angiogenesis via blockage of VEGF binding to VEGFR-2. The pathophysiological role of blood TIMP-3 in AD patients is still unknown. It is possible that the decreased blood TIMP-3 levels in AD patients may lead to imbalance between MMPs and TIMP and then cause BBB damage [54]. Although, the regulation of TIMP-3 in the pathology of AD is still not fully understood, we found that TIMP-3 levels are significantly lower in the plasma and CSF of AD patients. Decreased TIMP-3 level might be important for understanding the progression of AD. Our result also showed a decrease in TIMP-3 levels in the CSF of the MCI group compared with the control. The close correlations between TIMP-3 and Aβ_1-42_ levels in MCI suggested that TIMP-3 might be associated with AD progression.

The present findings should be interpreted considering some limitations. The patients recruited for their plasma TIMP-3 levels have different age and gender distributions between the control, MCI, and AD groups because of the limited sample size of the AGE cohort. Therefore, more replication studies with more participants are needed in the future. In conclusion, however, and despite these limitations, we observed that the plasma and CSF levels of TIMP-3 in patients with AD were decreased and associated with disease progression, suggesting that alterations of TIMP-3 may contribute to the understanding of AD progression.

## Figures and Tables

**Table 1:** Baseline characteristics of the population.

Features	Control	MCI	Dementia	*p*-Value
N (Male/Female)	115 (48/67)	71 (29/42)	65 (16/49)	
Age (years)	71.9 ± 0.43	73.05 ± 0.54	75.1 ± 0.75	0.001
Education	9.12 ± 0.47	6.18 ± 0.57	3.56 ± 0.55	<0.001
MMSE	27.21 ± 0.2	24.96 ± 0.35	16.0 ± 0.73	<0.001
CDR	0.043 ± 0.01	0.26 ± 0.02	1.12 ± 0.09	<0.001
Total CHOL	195.6 ± 3.2	190.3 ± 4.1	201.4 ± 4.5	0.306
TG	137.4 ± 7.2	127.9 ± 7.7	152.6 ± 11.5	0.287
HDL	43.4 ± 0.89	43.1 ± 1.46	44.5 ± 1.2	0.371
LDL	124.7 ± 3.0	121.5 ± 3.4	126.4 ± 3.8	0.599
Platelet	253.3 ± 6.6	268.0 ± 7.2	266.2 ± 11.3	0.229
Glucose	101.4 ± 2.0	102.3 ± 2.5	107.5 ± 5.1	0.483
vitB12	790.6 ± 57.3	699.2 ± 31.6	717.1 ± 41.3	0.903
TIMP-3 (ng/mL)	0.61 ± 0.06	0.40 ± 0.04	0.39 ± 0.05 ^a^	0.065

Values are mean ± SEM. MMSE, Mini-Mental State Examination; CDR, clinical dementia rating; SEM, standard error of the mean; Total CHOL, Total Cholesterol; TG, Triglycerides; HDL, High-density lipoprotein; LDL, Low-density lipoprotein; TIMP-3, Tissue inhibitor of metalloproteinase-3. *p*-values are for the Kruskal-Wallis test. Comparisons are assessed by Mann-Whitney U-tests. ^a^ Con: Dementia, *p* = 0.029.

**Table 2:** Correlations between plasma biomarkers and baseline characteristics.

Features	TIMP-3	
	Rho	*p*-Value
Age	−0.112	0.075
Education	0.094	0.14
MMSE	0.104	0.099
CDR	−0.153	**0.015**
Total CHOL	−0.105	0.096
Tg	0.008	0.902
HDL	−0.049	0.442
LDL	−0.142	**0.025**
Platelet	0.038	0.55
Glucose	−0.127	**0.046**
VitB12	0.079	0.21

The Spearman rank correlation coefficient test was used for assessment of correlation. Bold values are *p* < 0.05.

**Table 3:** CSF levels of Control Subjects, MCI and ADD patients.

Features	Control	MCI	ADD	*p*-Value
TIMP-3	2.26 ± 0.26	1.40 ± 0.29 ^1^	0.77 ± 0.11	0.004
Aβ_1-42_ (pg/mL)	1030.1 ± 32.6	766.3 ± 70.9 ^2^	401.3 ± 27.4 ^3^	<0.001
Total Tau (pg/mL)	224.3 ± 25.6	328.9 ± 69.5	480.8 ± 100.7	0.092
pTau (pg/mL)	50.3 ± 2.4	41.2 ± 4.5	81.5 ± 9.3 ^4^	0.001

Baseline characteristics of the population. Values are mean ± SEM. MCI, mild cognitive impairment; SEM, standard error of the mean. *p*-values are for the Kruskal-Wallis test. Comparisons are assessed with the Mann-Whitney U-test. ^1^ Con: MCI, *p* = 0.043; ^2^ Con: MCI, *p* = 0.003; ^3^ ADD: MCI, *p* = 0.002; ^4^ ADD: MCI, *p* = 0.001.

**Table 4:** Correlation between CSF biomarkers and CSF TIMP-3 levels.

Features	TIMP-3	
	Rho	*p*-Value
Age	−0.25	0.17
Aβ_1-42_	0.515	**0.004**
Total Tau	−0.337	0.069
pTau	−0.372	**0.047**

The Spearman rank correlation coefficient test was used for assessment of correlation. Bold values are *p* < 0.05.

## Data Availability

The data presented in this study are available on request from the corresponding author.
